# YKL-40 and genetic status of *CHI3L1* in a large group of asthmatics

**DOI:** 10.3402/ecrj.v2.25117

**Published:** 2015-09-16

**Authors:** Jakob W. Hansen, Simon F. Thomsen, Celeste Porsbjerg, Linda M. Rasmussen, Lotte Harmsen, Julia S. Johansen, Vibeke Backer

**Affiliations:** 1 Department of Respiratory Medicine, Bispebjerg Hospital, University of Copenhagen, Copenhagen, Denmark; 2 Department of Medicine, Herlev Hospital, University of Copenhagen, Copenhagen, Denmark

**Keywords:** asthma, rhinitis, YKL-40, CHI3L1, biomarker

## Abstract

**Background:**

Studies have shown a relationship between asthma, serum YKL-40, and the single nucleotide polymorphism (SNP) (−131 C/G, rs4950928) in the *CHI3L1* gene that codes for YKL-40. However, the findings differ. We studied the relationship between clinical asthma phenotypes, serum YKL-40, and SNP (−131 C/G, rs4950928).

**Methods:**

In this study, 1,137 patients with asthma, 415 with rhinitis only, and 275 non-asthmatic controls were included. Assessment included a clinical interview concerning the diagnosis of asthma, severity of asthma, and asthma treatment as well as clinical tests to assess asthma and rhinitis. Serum YKL-40 was measured, and genotyping for the SNP (−131 C/G) was conducted.

**Results:**

No significant difference in the serum concentration of YKL-40 was found between patients with asthma, patients with rhinitis, and non-asthmatic controls; however, YKL-40 was increased in patients with severe asthma. No association was found between the SNP (−131 C/G rs4950982) and the risk of having asthma (odds ratio = 0.90, *p*=0.4). Higher levels of serum YKL-40 were found in all subjects when comparing CC genotype to CG and GG genotypes (45 µg/L vs. 32 µg/L and 19 µg/L, *p*<0.0001).

**Conclusion:**

There was no association between polymorphisms of SNP (−131 C/G) and asthma. The highest serum YKL-40 concentrations were seen in severe asthmatics. Individuals with less severe asthma showed a smaller difference against controls, limiting its clinical usefulness. More research is needed to clarify the relationship between different asthma phenotypes, YKL-40, and *CHI3L1*.

Asthma is one of the most prevalent chronic diseases in young adults, and its frequency is increasing. Although environmental factors account for some of the increase in incidence (1, 2), genetic differences play a substantial role in asthma liability (3). Several studies have investigated the association between genetic variants and the expression of different asthma phenotypes (4); airway hyperresponsiveness (AHR); and airway inflammation including neutrophils, eosinophils, and macrophages (5). Taken together, this research indicates that multiple genes have a role in asthma (4, 6).

Serum YKL-40 is an inflammatory biomarker associated with disease activity and mortality in patients with diseases characterised by inflammation and tissue remodelling (7). The exact mechanism behind the increased mortality observed in patients with high serum YKL-40 is unknown (8). YKL-40 regulates vascular endothelial growth factor and has a role in inflammation (9, 10), cell proliferation and differentiation (11, 12), remodelling of the extracellular matrix (9), and protection against apoptosis (13).

YKL-40 is a chitinase-like protein and is secreted from cells in the airway mucosa, for example, neutrophils, macrophages, and airway epithelial cells (13, 14).

Chitinases are evolutionarily conserved proteins expressed in selected groups of asthma patients (15, 16). In addition, chitinases mediates both airway inflammation and airway remodelling in mouse models of asthma by a TH2 immune response (17, 18). One study has shown that YKL-40 promotes human bronchial smooth muscle cell proliferation and migration through PAR-2-, AKT-, ERK-, and p38-dependent mechanisms (19). They also found an effect of YKL-40 on increased cell migration was higher in bronchial smooth muscle cells from those with asthma compared with cells from healthy subjects (19). Serum concentrations of YKL-40 are associated with the severity of asthma and are inversely correlated with lung function; that is, forced expiratory volume in the first second (FEV1) (15), indicating that serum YKL-40 is important in the specific inflammatory phenotype of asthma.

It has been demonstrated that mechanical stress of bronchial epithelial cells potently induces *CHI3L1*, the gene encoding YKL-40, leading to increased secretion of YKL-40 in an EGFR and MEK1/2-dependent pathway (14). The *CHI3L1* gene (20) and the concentration of YKL-40 in serum have been found to be associated with a specific single nucleotide polymorphism (SNP) (−131 C/G, rs4950928) in the promoter region of the *CHI3L1* gene on chromosome 1q32.1. Ober et al. found a specific association between atopic asthma, serum concentration of YKL-40, and variation in this SNP in a population from a restricted geographic area (16). However, this genetic relationship has differed in other studies from other geographic areas (21). To date, studies are not conclusive regarding the genetic status of *CHI3L1* and the SNP (131 C/G), and more studies are needed to examine this gene and the clinical role of YKL-40 as a biomarker in asthmatic individuals.

Accordingly, we examined the association between the promoter SNP (131 C/G), serum YKL-40, and the presence of asthma in a large study of well-characterised Danish patients with mild to severe asthma.

## Materials and methods

### Design

All subjects completed a self-administered questionnaire concerning asthma and rhinitis symptoms, and quality of life and underwent lung-function tests before being clinically examined. Lung function was measured and skin prick test was performed, after which a bronchial provocation test with methacholine and a reversibility test with inhaled beta_2_-agonist was done. Blood was drawn, and serum and buffy coat were subsequently collected and stored at −80°C, where the YKL-40 level is known to be stable for at least 15 years (22). Medical history was gathered by a respiratory physician through a questionnaire-based structured interview. Buffy coats were shipped to deCode Genetics, Iceland, for genotyping. Serum concentrations of YKL-40 were measured at Herlev University Hospital, Copenhagen, Denmark.

### Participants

Data concerning *CHI3L1* (−131 C/G, rs4950928) and serum YKL-40 were recorded in 1,921 subjects who were examined consecutively in 2000 and 2001 at the Respiratory Research Unit, Bispebjerg University Hospital, Copenhagen, and in 2005 at Aalborg University Hospital, Denmark (23–27). All subjects underwent the same examinations. Buffy coats were obtained from 1,890 (98%) subjects, and *CHI3L1* genotyping was performed. In 1,041 (55%) subjects, sufficient serum was stored for measurement of serum YKL-40.

### Study population

Of the 1,921 subjects, 1,137 were classified as having asthma and 415 as having rhinitis only; 275 were classified as non-asthmatic controls as they had normal lung function, no atopy, and a negative methacholine provocation and reversibility test, although they complained of mild cough or wheeze (23–28). The remaining 94 subjects were excluded from further analysis due to the following reasons: 75 were diagnosed as symptomatic smokers, with symptoms of coughing for three consecutive years, indicating chronic bronchitis; six were diagnosed with chronic obstructive pulmonary disease (COPD); nine were diagnosed with asymptomatic atopy; three subjects with non-atopic nasal symptoms had asymptomatic AHR; and one was excluded because of missing data.

Patients and healthy subjects received information verbally and in writing and gave written consent before enrolment. The study was approved by the local ethics committee of Copenhagen, Denmark, and was conducted according to the Declaration of Helsinki.

### Pre-medication

Prior to asthma testing, those participants who were taking medication for asthma and allergy were asked not to use theophylline or antihistamines for at least 24 hours. Short-acting inhaled bronchodilators should be discontinued for 12 hours and long-acting for 24 hours. Participants were allowed to continue use of any inhaled or oral corticosteroids. Any antihistamines were discontinued for at least 3 days.

### Basic assessment

All participants with asthma were classified according to the Global Initiative for Asthma (GINA) guidelines, on the basis of the frequency of symptoms and level of lung function (29) (FEV1% of predicted): 1) intermittent: symptoms <1 time a week, nighttime symptoms <2 times a month, FEV1 > 80% predicted; 2) Mild persistent: symptoms >1 time a week but <1 time per day, nighttime symptoms >2 times a month, FEV1 >80% predicted; 3) Moderate persistent: daily symptoms, nighttime symptoms >1/week, or FEV1 60–80% predicted; and 4) Severe: continuous symptoms, frequent nighttime symptoms, or FEV1 < 60%.

### Exclusion criteria

Patients were excluded if they had respiratory illnesses other than asthma (e.g. sarcoidosis, COPD). Patients with a respiratory tract infection had their provocation test postponed for 6 weeks. Patients with asymptomatic AHR were excluded from further analysis.

### Medical history

All subjects completed self-administered questionnaires before clinical and physical tests. Subjects were asked about respiratory and allergic symptoms [within the preceding 4 weeks and at any time (ever asthma)], use of medication, hospital referrals, and visits to a general practitioner or a specialist. The interview questions about asthma were adapted from studies by the American Thoracic Society, Division of Lung Disease of the National Heart, Lung and Blood Institute (30). Lifetime consumption of tobacco was measured in pack years (tobacco consumption [g/day]/20×duration of smoking [years]). Height and weight were measured in all participants, and body mass index (BMI) was calculated (weight [kg]/height [m]^2^).

### Diagnosis of asthma

The diagnosis of asthma was based on the presence of respiratory symptoms and AHR to inhaled methacholine <8.0 µmol, or at least 250 ml increase in FEV1 after bronchodilator challenge. Subjects were also defined as having asthma if they had a doctor's diagnosis of asthma and daily use of systemic steroid, inhaled steroid, fixed combination of inhaled corticoid steroids or long-acting beta agonists, or use of inhaled beta_2_-agonist after classical exercise-induced asthma symptoms. Subjects with seasonal symptoms of asthma, tested off-season, were included as having asthma if they reported recurrent seasonal symptoms and a relevant atopy or a history of rhinitis. Non-smokers with classical respiratory symptoms suggestive of asthma and signs of mild AHR, with a percentage fall in FEV1 of 10% or more after inhalation of 8.0 µmol methacholine, were classified as mildly asthmatic.

### Pulmonary function test and methacholine challenge test

Spirometry was performed on a 7-L dry wedge spirometer (Vitalograph^®^) in accordance with the European Respiratory Society, and the percentage of predicted normal values of FEV1 (FEV1% of predicted) and forced vital capacity (FVC) (FVC% of predicted) and the FEV1/FVC ratio was calculated (31).

Airway responsiveness to inhaled methacholine was measured according to the method of Crapo et al. (32) in all patients with predicted FEV1 >70%. The dose resulting in a 20% fall in FEV1 (PD_20_) was calculated, and the response dose-ratio (RDR) was calculated as the decline in FEV1 from inhaled saline divided by the highest dose of methacholine administered (33). A constant of 5 was added to all RDRs to eliminate negative and zero values. Logarithmically transformed values of RDR were used for analysis. Measurement of FEV1 was repeated 15 minutes after administration of 2 mg terbutaline in those with FEV1 <70%, or 15 minutes after the last inhalation of methacholine in those with either symptoms or a significant decrease in FEV1 (i.e. 20%).

### Skin prick test

Skin prick test was performed using standard dilutions of 10 common aeroallergens. The allergens used were birch, grass, mugwort, horse, dog, cat, house dust mite (*Dermatophagoides pteronyssinus* and *Dermatophagoides farinae*), and mould (*Alternaria iridis* and *Cladosporium herbarum*). The positive reference was histamine 10 mg/mL in 50% glycerol, whereas the negative reference was 50% glycerol (Soluprick SQ system; ALK Albelló, Hoersholm, Denmark). A positive result (atopy) was defined as a positive reaction to at least one allergen, and a reaction was considered positive if the mean wheal diameter was at least 3 mm. Subjects with asthma concomitant with a positive skin prick test were regarded as atopic (34).

### Genotyping of the promoter SNP (−131 C/G, rs4950928) in the *CHI3L1* gene

Within 2 hours after blood was drawn, serum and buffy coat were stored at −80°C. Genotyping was done at deCODE Genetics (Reykjavik, Iceland), using the Centaurus Nanogen, (Bothell, WA, USA) genotype platform.

### YKL-40 analysis

Serum concentration of YKL-40 was determined in duplicates, in samples frozen for 3–8 years at 80°C, by a commercial two-site, sandwich-type enzyme-linked immunosorbent assay (ELISA) (Quidel Corporation, San Diego, California) using streptavidin-coated microplate wells, a biotinylated-Fab monoclonal capture antibody, and an alkaline phosphatase-labelled polyclonal detection antibody. The recovery of the ELISA was 102% and the detection limit was 10 µg/L. The intra-assay coefficients of variations were 5% (at 40 µg/L), 4% (at 104 µg/L), and 4% (at 155 µg/L). The inter-assay coefficient of variation was <6% (22).

### Statistical analysis

Mean and standard deviations (±SD) were calculated for normally distributed variables, whereas median and interquartile ranges were used to describe variables with a non-Gaussian distribution (serum YKL-40). For the continuous variables, data were analysed by ANOVA followed by the two-sample t-test to compare the groups or paired t-test to compare changes within the group. The Chi-squared test for unpaired data was used for the categorical variables. The Kruskal-Wallis and Mann-Whitney U tests were applied for analysis of non-parametric ordinal variables. Odds ratio (OR) with a 95% confidence interval was calculated to analyse differences between the alleles, genotypes, and phenotypes. Two-sided *p*-values and allele-specific OR for each individual allele assuming a multiplicative model were calculated standard likelihood ratio statistics as implemented in the NEMO software (35). Allele and haplotype frequencies were estimated by maximum likelihood and calculated directly for the observed data, and tests of differences between cases and controls were performed using a generalised likelihood ratio test (35).

A two-sided *p*-value below 0.05 was considered significant. All analyses were performed using the statistical pack SPSS version 17.0 (Chicago, Illinois).

## Results

The clinical characteristics of the patients with asthma or rhinitis and the controls are listed in Table 1. All those with asthma had lower lung function compared with controls (FEV1% of predicted 95.0% vs. 103%, *p*<0.001) and a higher level of airway responsiveness (logRDR 0.85 vs. 0.82, *p*<0.001). No difference was found in airway obstruction between asthma and absence of asthma (0.82 vs. 0.81, *p*<0.092). Of the patients with asthma, 343 had intermittent asthma, 312 had mild persistent asthma, 280 had moderate asthma, and 202 had severe asthma.

**Table 1:** Characteristics of cases and controls

Characteristic	Asthma	Asthma with rhinitis	Rhinitis	Controls	*p* One way ANOVA
No	271	866	415	275	
Female^a^	210 (78)	563 (65)	216 (52)	155 (56)	<0.001
Age (years)^b^	30 (29–30)	30 (30–31)	32 (31–33)	33 (32–34)	<0.001
BMI (kg/m^2^)^b^	25 (24–26)	25 (25–26)	25 (25–26)	25 (25–26)	NS
FEV1,% of predicted^b^	94.9 (93.2–96.6)	93.7 (92.8–94.7)	100.0 (98.8–101.1)	103.0 (101.5–104.4)	<0.001
FVC,% of predicted^b^	97.2 (95.5–98.8)	96.2 (95.3–97.0)	97.6 (96.2–98.9)	97.0 (94.7–99.3)	NS
FEV1/FVC^b^	0.82 (0.81–0.83)	0.81 (0.80–0.81)	0.84 (0.83–0.84)	0.83 (0.82–0.83	<0.001
logRDR^b^	0.77 (0.70–0.84	0.88 (0.84–0.92)	0.30 (0.25–0.35)	0.16 (0.07–0.24)	<0.001
CC genotype^a^	161 (61)	519 (61)	243 (59)	167 (64)	NS
CG genotype^a^	97 (37)	297 (35)	148 (36)	87 (33)	NS
GG genotype^a^	5 (2)	39 (5)	19 (5)	8 (3)	NS
Serum YKL-40 (µg/L)^c^	40 (14–193)	40 (10–280)	38 (11–315)	43 (14–195)	NS (Kruskal-Wallis)

RDR, response dose-ratio; NS, non-significant.

a Values are number (%).

b Values are median (95% CI).

c Values are range (min–max).

The group of controls included fewer females than the entire group of patients with respiratory disease (56% vs. with 64%, *p*=0.05). Age differed slightly in the control group, but BMI was similar to the entire group of asthmatic patients. The controls showed no signs of airflow limitation, AHR, or atopy.

### Serum YKL-40 and asthma

**Fig. 1:**
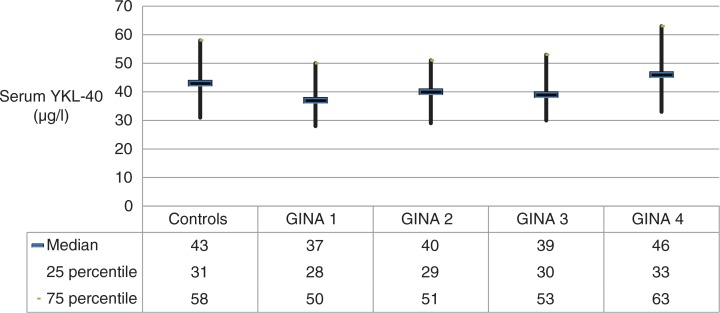
Serum concentrations of YKL-40 (µg/L) in patients with asthma in relation to GINA classification.

There were no differences in serum concentrations of YKL-40 across the entire group of patients with asthma and controls median (min–max) 40 µg/L (10–280 µg/L) versus 43 µg/L (14–195 µg/L), *p*=0.106, nor between patients with asthma and asthma with rhinitis, or between patients with rhinitis and controls (Table 1). In patients with asthma, serum YKL-40 was increased in those with the most severe asthma compared with the remaining patients (GINA 1–4); median (25–75 percentile) serum YKL-40: intermittent 37 µg/L (28–50 µg/L); mild persistent 40 µg/L (29–51 µg/L); moderate persistent 39 µg/L (30–53 µg/L); and severe persistent 46 µg/L (33–63 µg/L), *p*=0.009) (Fig. 1).

### SNP (−131 C/G, rs4950928) in *CHI3L1* and asthma

There was no association between having the C allele in the SNP (131 C/G) and the risk of having asthma (OR = 0.90, *p*=0.37). We further analysed the association between SNP (131 C/G) and different phenotypes of asthma (asthma only, asthma and rhinitis), as well as rhinitis only, and no associations were found (Table 2).

**Table 2:** Relationship between the *CHI3L1* gene and different phenotypes

		*CHI3L1* rs4950928 C allele Odds ratio	*p*	Allele frequency	Total allele frequency
Asthma	Cases	0.899	0.374	0.78	0.79
	controls			0.80	
Rhinitis	Cases	0.848	0.228	0.77	0.79
	controls			0.80	
Asthma and	Cases	0.881	0.302	0.78	0.79
rhinitis	controls			0.80	

### Serum concentrations of YKL-40, SNP (−131 C/G, rs4950928) in *CHI3L1* and asthma

In a univariate analysis, the serum concentration of YKL-40 was associated with SNP (131 C/G) (*p*<0.001), asthma severity (GINA 1–4, *p*<0.01), BMI (*p*<0.001), and smoking indicated by pack year (*p*<0.001). In contrast serum YKL-40 was not associated with asthma as a dichotomous variable (yes/no), FEV1% predicted, FEV1/FVC ratio, logRDR, sex, age and rhinitis.

When factors associated with YKL-40 were included in a multivariate regression analysis, the associations between serum YKL-40 and SNP (131 C/G) (*p*<0.001), smoking (*p*<0.001), asthma severity (GINA 1–4, *p*=0.001), and BMI (*p*<0.01) remained statistically significant.

Differences in SNP (131 C/G) in the promoter region of *CHI3L1* explained 7.9% of the variance in serum concentrations of YKL-40.

The highest serum concentration of YKL-40 was found in patients with asthma, independent of their status of rhinitis, with the SNP −131 CC genotype, and the lowest serum concentration of YKL-40 in patients with asthma with the SNP −131 GG genotype (median [min–max]: 46 µg/L [16–280 µg/L] vs. 19 µg/L [10–44 µg/L], *p*<0.0001) (Table 3).

**Table 3:** Relationship between the genotype on rs4950928 (SNP −131 CG) and serum YKL-40

	Serum YKL-40 µg/L				
	
*CHI3L1* rs4950928 genotype	Total (*n*=1025)	Asthma (*n*=143)	Asthma + Rhinitis (*n*=524)	Rhinitis (*n*=190)	Controls (*n*=168)
CC	45 (16–315)	48 (18–193)	46 (16–280)	42 (18–315)	49 (24–195)
CG	32 (14–256)	32 (17–63)	32 (14–256)	32 (15–123)	32 (14–118)
GG	19 (10–44)	26 (14–32)	19 (10–44)	14 (11–22)	21 (15–25)

Values are median (min–max). *P*<0.001 between groups.

## Discussion

In this large study of well-characterised Danish patients with mild to severe asthma, we found that patients with the *CHI3L1* (131 CC, rs4950928) genotype had higher serum concentrations of YKL-40 compared with patients with the *CHI3L1* (−131 GG, rs4950928) genotype. This was also the case in the healthy subjects. No association was found between the *CHI3L1* (−131 C/G, (rs4950928) genotype and asthma. Furthermore, serum YKL-40 was significantly higher in those with severe asthma (GINA 4) compared with healthy subjects and patients with GINA 1–3. These findings indicate that the *CHI3L1* (−131 CC, rs4950928) genotype and serum YKL-40 are not further implicated in the pathogenesis of asthma, but YKL-40 may be a potential biomarker of inflammation related to the severity of asthma—the more severe the asthma, the higher the serum YKL-40.

Our findings are in contrast to those of two previous studies that showed an association between the *CHI3L1* (−131 C/G, rs4950928) genotype and the risk of asthma (15, 21).

Ober et al. (16) found an association with homozygosity of the CC allele and atopic asthma in a highly selected population. A more recent study of children and young adults (36) tried to replicate the work of Ober et al. (16) and found an association of homozygosity of the CC allele and hospital admissions due to asthma exacerbations, and could not replicate the relationship between the SNP rs4950928 and lung function. In a large population study of 6,514 individuals, Rathcke et al. found an association with GG homozygotes and risk of asthma (21).

We studied a large group of patients who were interviewed by a respiratory specialist and subsequently had their asthma diagnosis defined by respiratory symptoms and a positive asthma test, securing a relatively uniform asthma population. The patients were unselected and invited from a nationwide pool of Danish patients from an area with approximately five million inhabitants; all had mild to severe asthma. Based on the present findings, it is difficult to be conclusive about a connection between the SNP (−131 C/G, rs4950928) in the *CHI3L1* gene and the risk of having asthma.

The differences in findings between our study and the aforementioned studies could be due to differences in study design. Our study included a large group of Danish asthma patients, which is also the case in the other Danish population-based Inter99 study of 6,514 individuals, including 540 subjects with self-reported asthma (21). However, those patients were diagnosed based on a questionnaire, whereas our patients were diagnosed by a specialist and all underwent asthma tests. This makes these two studies different and could explain why the findings are different. Furthermore, Ober et al. (16) had a highly selected study population, which was smaller than ours, from a restricted area in the United States.

We also analysed the relationship between the *CHI3L1* (−131 C/G, rs4950928) genotype and serum concentrations of YKL-40 and found the highest serum YKL-40 in patients with the *CHI3L1* (−131 CC) genotype. This is in accordance with the findings of Ober et al. (16). We demonstrated that the *CHI3L1* (−131 C/G, rs4950928) genotype explained 7.9% of the variation in serum YKL-40, and this is similar to the results by Ober et al. where 9.4% of the variation in serum YKL-40 was explained by differences in the *CHI3L1* (−131 C/G, rs4950928) genotype.

These similarities are important because the clinical asthma phenotype could be different due to different perceptions of disease, whereas a relationship between serum concentration of YKL-40 and genes provides the possibility of using serum biomarkers, rather than genes, in future planning of tailored medication for patients with asthma.

Elevated serum concentrations of YKL-40 are seen in patients with several different diseases, but all are characterised by inflammation and ongoing tissue remodelling, for example, ischemic cardiovascular diseases, cancer, diabetes, COPD, rheumatoid arthritis, inflammatory bowel disease, pneumonia, and liver fibrosis (37–41). This suggests that serum concentrations of YKL-40 may also be a biomarker in patients with different types of lung disease, including asthma. The relationship between the serum concentration of YKL-40 and the severity of asthma determined by the GINA classification found in the present cohort of patients with asthma is in accordance with a recent study by Tang et al., which showed higher serum YKL-40 in patients with more severe or uncontrolled asthma (42). Idiopathic pulmonary fibrosis has also been associated with increased serum concentration of YKL-40 (43). This indicates that the increased serum concentration of YKL-40 in patients with the most severe asthma might be due to increased levels of fibrosis with ongoing tissue remodelling and neutrophil inflammation in the airways (43). Bara et al. have shown that YKL-40 plays a role in bronchial remodelling, through migration and proliferation of bronchial smooth muscles (19). YKL-40 concentrations in serum tend to be high when patients have active inflammatory diseases. These patients may need immediate treatment, and the overlap between severities could indicate that serum YKL-40 would be a better biomarker for significant activity rather than perception of symptoms, which is the basis of the GINA classification.

In the present study, patients with asthma had a lower serum concentration of YKL-40 compared with patients with the same degree of asthma seen in other studies (15, 16). Even the most severe cases of asthma in our study had lower levels of serum YKL-40 than did controls in the other studies investigating serum YKL-40 in asthmatic individuals (15, 16, 42) Those with severe asthma in two other studies had almost a two-fold increase in serum YKL-40 compared with their counterparts in our study. This suggests that those with severe asthma in our population had lower inflammation either because of better treatment or simply because of another inflammatory pattern. Lastly, the disparity could stem from differences in the selected population regarding smoking, age, and BMI. For example, our subjects were younger and had a lower BMI compared with those in the study by Chupp et al. (15). Moreover, among those with severe asthma, there might be a difference in inflammatory cells, which are known not to be uniform, and this could affect the expression of YKL-40 (44) in this group of patients. Clearly, the association between YKL-40 and different phenotypes of asthma needs to be investigated more thoroughly, and the role of YKL-40 as a biomarker of asthma is still undetermined and its rightful use in asthma has yet to be found.

The strength of our study is its relatively large population size, the identical clinical classification of all patients with asthma, and the large geographic area from which the population was recruited. A limitation is the relatively small number of controls compared with the number of patients with asthma. Although some of our controls reported respiratory symptoms, a respiratory specialist designated them as controls because they had no signs of reversible obstructive lung disease.

In conclusion, in a large group of patients with asthma, we found that serum concentrations of YKL-40 were only slightly increased in those with the most severe asthma. The clinical use of serum YKL-40 in asthmatic individuals remains largely undetermined. Since serum concentrations of YKL-40 seem to reflect ongoing angiogenesis and tissue remodelling (9, 10), serum YKL-40 may be a biomarker of both acute and chronic inflammatory activity. We encourage future studies to investigate the differences in the type of airway inflammation and the relationship with serum YKL-40, as differences in asthma phenotypes relate to bronchial inflammation as well as the number of inflammatory cells (14, 45). Serum YKL-40 might be a useful biomarker that could indicate the need for treatment, when measured in the correct phenotype of patients. A study investigating the changes in serum YKL-40 in patients with asthma who are uncontrolled and who are undergoing treatment could provide important biological information.

There was no increased risk of asthma associated with different genotypes on the SNP (−131 C/G, rs4950928) in the *CHI3L1* gene in our study. This finding cannot stand alone but should be seen as a contribution to the discussion about the genetic role of *CHI3L1* in asthma. The literature is conflicting regarding the presence of the *CHI3L1* gene and an increased risk of asthma, and the present study supports no association between the SNP rs4950928 and development of disease. Lastly, we showed a connection between serum concentrations of YKL-40 and the *CHI3L1* (−131 C/G, rs4950928) genotype, which supports findings from other studies.
